# Sickness absenteeism among health care workers in the municipal public service of Chapecó-SC, in the period 2015-2018

**DOI:** 10.47626/1679-4435-2022-962

**Published:** 2024-08-05

**Authors:** Diego Pozzer, Adriana Remião Luzardo, Joanna d’Arc Lyra Batista, Paulo Roberto Barbato

**Affiliations:** 1 Secretaria Municipal de Saúde, Chapecó, SC, Brazil; 2 Curso de Pós-graduação Lato Sensu em Saúde Coletiva, Universidade Federal da Fronteira Sul, Chapecó, SC, Brazil

**Keywords:** absenteeism, occupational health, public sector, epidemiology, absenteísmo, saúde do trabalhador, setor público, epidemiologia

## Abstract

**Introduction:**

Absenteeism of health workers is important because it interferes with the quality of care provided to patients.

**Objectives:**

To characterize the absenteeism-illness of workers in the municipal public health network in Chapecó, SC, Brazil (2015-2018) and test the association of two or more absences in the year with the study variables.

**Methods:**

A crosssectional study was conducted, and the variables studied were sex; age group; professional category; acting time; International Classification of Diseases and Related Health Problems, and sick leave. Descriptive analysis were performed, the factors associated with the outcome were tested, and the prevalence ratios were calculated with their respective 95% confidence intervals using Poisson regression.

**Results:**

A total of 1,695 professionals on sick leave were identified, with a higher prevalence of women (89.40%), in the 30-39 age group (33.41%), the majority with one sick leave per year (61.24%), from 3 to 9 days (47.67%). Community health workers were the category that most frequently had sick leaves (27.15%). In the years studied, there were 2,795 sick leaves (657 employees with more than one sick leaves). Musculoskeletal disorders were the main causes (21.80%) and the highest prevalence was dorsopathies (57.60%). Working for 21 years or more had a 49% higher prevalence ratio for two or more sick leaves per year, compared to having been working for up to 5 years.

**Conclusions:**

The study allowed us to characterize absenteeism-illness among workers in the healthcare services in Chapecó, SC. The results may constitute indicators of human resource management and foster strategies to promote healthy environments, prevention of diseases and injuries, and rehabilitation.

## Abstract (pt)

**Introdução:**

O absenteísmo de trabalhadores da saúde é importante por interferir na qualidade da assistência prestada aos pacientes.

**Objetivos:**

Caracterizar o absenteísmo-doença dos trabalhadores da rede pública municipal de saúde de Chapecó, no estado de Santa Catarina, no período de 2015 a 2018 e testar a associação de dois ou mais afastamentos no ano com as variáveis de estudo.

**Métodos:**

Foi realizado um estudo transversal, e as variáveis estudadas foram sexo, faixa etária, categoria profissional, tempo de atuação, Classificação Internacional de Doenças e tempo de afastamento. Foram realizadas análises descritivas, testados os fatores associados ao desfecho e calculadas as razões de prevalência com os respectivos intervalos de confiança de 95% por meio da regressão de Poisson.

**Resultados:**

Foram identificados 1.695 profissionais afastados por doença, com maior prevalência do sexo feminino (89,40%), na faixa de 30 a 39 anos (33,41%), e a maioria apresentou um afastamento ao ano (61,24%), de 3 a 9 dias (47,67%). Os agentes comunitários de saúde foram a categoria com mais afastamentos (27,15%). Nos anos estudados, houve 2.795 afastamentos (657 servidores com mais de um afastamento). As doenças osteomusculares foram as principais causas identificadas (21,80%), com maior prevalência de dorsopatias (57,60%). Atuar há 21 anos ou mais apresentou razão de prevalência 49% maior para dois ou mais afastamentos no ano em comparação a estar atuando por até 5 anos.

**Conclusões:**

O estudo permitiu caracterizar o absenteísmo-doença entre trabalhadores da rede municipal de saúde de Chapecó. Os resultados encontrados poderão constituirse como indicadores de gestão de recursos humanos e fomentar estratégias de promoção de ambientes saudáveis, prevenção de doenças e agravos e reabilitação.

## INTRODUCTION

Work has always played an important role in human life because it is not only a means of production for society, but also a source of personal fulfillment. However, as capitalism and industry have expanded, work processes have undergone major changes in terms of their division and the ways in which they are organized, requiring a high degree of dynamism and great physical and psychological effort, sometimes exceeding the limits of the worker’s capacity. As a result, the very work that dignifies human beings and gives them personal fulfillment and social contribution can also lead to a series of aggravating factors for their integrality and health.^1,2^

In this scenario, health professionals are among the categories most likely to be at risk of accidents, illness, and withdrawal from work due to the high workload, exposure to the environment, unhealthy conditions, demands for productivity, and physical and mental imbalance. Absenteeism is one of the consequences of the relationship between these risks; sick leave is the most prevalent and has the greatest impact on workers’ lives.^3^

According to the International Labor Organization, sickness absenteeism is defined as absence from work due to illness or accidental injury, whether or not related to the work environment, except in cases of pregnancy or imprisonment.^4^

Sickness absenteeism is important in public health, as it reflects the health status of workers and has important economic impacts, as it interferes with production and increases costs for companies and social security, and reduces the efficiency and quality of work.^5^

Absenteeism among healthcare workers is a problem of great concern, as it disrupts the service and adds to the workload of other team members, wearing down the workers who remain at their jobs and, consequently, increasing the number of absences. As a result, absenteeism causes complex and costly administrative problems by substantially increasing operational costs, and indirectly interfering with the quality of care provided to patients.^6^

It is therefore necessary to take a broad view of the problem in order to understand the phenomenon of sick leave, thus encouraging discussion in the public administration sectors. In view of this, this study aimed to describe sickness absenteeism among public health workers in the municipality of Chapecó, SC, Brazil, from 2015 to 2018, and to test the association between having been absent twice or more during the year and the study variables.

## METHODS

This quantitative, cross-sectional, and analytical study used retrospective and secondary data on the occurrence of sickness absenteeism among local civil servants.

This study was conducted in the municipality of Chapecó, a town in the west of Santa Catarina, in the southern region of Brazil, with an estimated population of 224,013 inhabitants.^7^ We collected data from the sick leave records of the Serviço de Atendimento à Saúde do Servidor Público Municipal (SASSM) and the Human Resources (HR) department of the Secretaria Municipal de Saúde (SESAU).

According to Complementary Law No. 360, article 2,^8^ SASSM aims to “promote the health and protect the integrity of civil servants in the workplace, ensuring their physical and mental health, and monitoring their health problems”.

The sample consisted of health workers from the municipality of Chapecó who were absent from work due to illness between 2015 and 2018. It should be noted that the last 4 months of 2018 were not included in the study, as the service had not yet calculated the information for this period. Cases of sick leave of 3 days or more with an illness duly confirmed according to the International Classification of Diseases 10th revision (ICD-10) were included. Absences due to maternity leave and to accompany a sick family member were excluded from the study.

The variables included in the study were sex (male and female); age (in years), subsequently organized into age groups; professional category, with length of professional experience in the health department (in years); ICD of the disease; and length of absence (in days).

Data were collected in October 2020, using information from the medical reports registered with SASSM, according to the ICD-10 criteria. The social and demographic data not found in the SASSM records were supplemented with information available in the HR department based solely on the civil servant registration number.

The data were organized in a Microsoft Office Excel 365 spreadsheet. We performed descriptive analyses using measures of central tendency and dispersion and absolute and relative frequencies of the variables. In order to identify the factors associated with the outcome (having two or more sick leaves in a year), the prevalence was calculated with the respective 95% confidence intervals (95%CI), and the association was tested using the chi-squared test, which was significant when its p-value was ≤ 0.05. Prevalence ratios (PR) and the respective 95%CI were also calculated using Poisson regression for the statistically significant variables. The data were analyzed using Stata^®^ software, version 12.1.

This study was authorized by SESAU and approved by the Research Ethics Committee of the Universidade Federal da Fronteira Sul (UFFS), according to opinion No. 3.803.009, of January 17, 2020. This study complied with the ethical precepts of Resolution No. 466/2012 of the Conselho Nacional de Saúde (Brazil National Health Council), which regulates the participation of human beings in research.

## RESULTS

From January 1, 2015 to August 31, 2018, SASSM recorded a total of 1,695 civil servants on sick leave from the public health network.

The average age of civil servants on sick leave between 2015 and 2018 was 41.1 years (95%CI 40.641.6; standard deviation [SD] 10.1 years), with a median age of 41 years. The youngest civil servant was 17 years old and the oldest was 77 years old.

The average length of service in the public health service for workers on sick leave during the period was 7.1 years (95%CI 6.7-7.4; SD 6.8 years), with a median of 5 years of work; the worker on sick leave with the shortest working time had worked for 1 month and the longest working time was 30 years in the health service.

Table 1 shows the profile of the staff on sick leave, with a predominance of females (89.40%), aged between 30 and 39 (33.41%), and working for less than 5 years (52.66%). The majority of civil servants had only one sick leave in a year (61.24%), and most sick leaves were between 3 and 9 days (47.67%).

**Table 1:** Profile of employees of the Secretaria Municipal de Saúde (Municipal Health Department) of Chapecó, SC, Brazil on sick leave (n = 1,695) between 2015 and 2018

Variables	n	%
Sex Male	180	10.60
Female	1,515	89.40
Age group (years) Up to 29	222	13.11
30-39	566	33.41
40-49	531	31.35
50-59	314	18.54
60 or older	61	3.60
Length of service (years) Up to 5	891	52.66
6-10	388	22.93
11-20	313	18.50
21 or more Total annual sick leave	100	5.91
Once	1,038	61.24
Twice or more Annual sick leave (days)	657	38.76
3-9	808	47.67
10-30	526	31.03
31 or more	361	21.30

Table 2 shows the relative distribution of sick leave according to professional category. Community health workers (CHWs) had the highest prevalence of sick leave (27.15%), followed by nursing assistants (25.97%) and doctors (9.03%). The professional categories of 12 workers on sick leave were not recorded.

**Table 2:** Absolute and relative frequencies of sick leaves according to the professional category of employees of the Secretaria Municipal de Saúde (SESAU) of Chapecó, SC, Brazil, between 2015 and 2018

Professional category	n	%
Community health worker	457	2715
Nursing assistant	437	25.97
Physician	152	9.03
Endemic disease control worker	150	8.91
Nurse	115	6.83
Dental office assistant	51	3.03
Dental surgeon	48	2.85
Internal services assistant	43	2.55
Pharmacist	37	2.20
Administrative assistant	35	2.08
Nursing technician	33	1.96
Psychologist	29	1.72
Driver	26	1.54
Physiotherapist	15	0.89
Nutritionist	9	0.53
Social worker	6	0.36
Occupational therapist	6	0.36
Health inspector	6	0.36
Administration technical assistant	6	0.36
Laboratory technician	5	0.29
Speech-language pathologist	4	0.24
Administration technician	4	0.24
Biologist	3	0.18
Physical education practitioner	3	0.18
Laboratory assistant	3	0.18
Total	1,683	100.00

In the years studied, a total of 2,795 sick leaves were recorded, and 657 civil servants were absent more than once. The mean annual sick leave was 30.7 days (95%CI 28.0-33.4), SD 56.7 days and median 10 days.

According to the ICD-10 chapters, the main causes of sick leave were included in chapter XIII (diseases of the musculoskeletal and connective system, codes M00-M99), comprising 21.8% of the records. Next, the most prevalent absences were due to mental and behavioral disorders (chapter V - codes F00-F99), with 16.8% of cases, and diseases of the respiratory system (chapter X - codes J00-J99), with 9.1% of all sick leaves. Table 3 shows the details of sick leave as per the ICD-10 chapters.

**Table 3:** Illness-related sick leave, according to the chapters of the International Classification of Diseases 10th revision, among employees of the Secretaria Municipal de Saúde (Municipal Health Department) of Chapecó, SC, Brazil, from 2015 to 2018

Chapter	Description	Codes	n (%)
I	Certain infectious and parasitic diseases	AOO-B99	90 (3.2)
II	Neoplasms	C00-D48	68(2.4)
III	Diseases of the blood and blood-forming organs and certain disorders involving the immune mechanism	D50-D89	5 (0.2)
IV	Endocrine, nutritional and metabolic diseases	EOO-E90	14 (0.5)
V	Mental and behavioral disorders	FOO-F99	471 (16.8)
VI	Diseases of the nervous system	G00-G99	44 (1.6)
VII	Diseases of the eye and adnexa	H00-H59	174 (6.2)
VIII	Diseases of the ear and mastoid process	H60-H95	28 (1.0)
IX	Diseases of the circulatory system	IOO-I99	92 (3.3)
X	Diseases of the respiratory system	J00-J99	256 (9.1)
XI	Diseases of the digestive system	K00-K93	156 (5.6)
XII	Diseases of the skin and subcutaneous tissue	L00-L99	36 (1.3)
XIII	Diseases of the musculoskeletal system and connective tissue	M00-M99	609(21.8)
XIV	Diseases of the genitourinary system	N00-N99	138(4.9)
XV	Pregnancy, childbirth and the puerperium	000-099	186 (6.6)
XVI	Certain conditions originating in the perinatal period	P00-P96	2 (0.1)
XVII	Congenital malformations, deformations and chromosomal abnormalities	Q00-Q99	5 (0.2)
XVIII	Symptoms, signs and abnormal clinical and laboratory findings, not elsewhere classified	R00-R99	83 (3.0)
XIX	Injury, poisoning and certain other consequences of external causes	S00-T98	226 (8.1)
XX	External causes of morbidity and mortality	V01-Y98	19 (0.7)
XXI Total	Factors influencing health status and contact with health services	Z00-Z99	93(3.4) 2,795 (100.0)

Although the highest prevalence of sick leave was related to Chapter XIII, when looking at the diagnoses in absolute numbers, dorsopathies were diagnosed 351 times, mood/depression disorders 342 times, and acute respiratory infections 210 times. Table 4 shows the diagnoses which led to sick leave according to the three ICD-10 chapters most prevalent in sick leave.

**Table 4:** Illness-related causes of sick leave among Secretaria Municipal de Saúde (Municipal Health Department) employees in Chapecó, SC, Brazil, between 2015 and 2018

Disease categories and ICD codes	n	%
Diseases of the musculoskeletal system and connective tissue (M00-M99)		
Arthropathies (MOO-M25)	114	18.7
Dorsopathies (M40-M54)	351	57.6
Disorders of muscles (M60-M63)	8	1.3
Disorders of synovium and tendon (M65-M68)	16	2.6
Other soft tissue disorders (M7O-M79)	118	19.4
Osteopathies and chondropathies (M80-M94)	2	0.4
Mental and behavioral disorders (FOO-F99)		
Organic anxiety disorders (F06)	1	0.2
Mental and behavioral disorders due to psychoactive substance use (F1O-F19)	11	2.3
Schizoaffective disorders (F25)	3	0.6
Mood [affective] disorders (F3O-F39)	342	72.6
Neurotic, stress-related disorders (F4O-F48)	112	23.8
Specific personality disorders (F60)	2	0.5
Diseases of the respiratory system (JOO-J99)		
Other acute lower respiratory infections (JOO-J22)	210	82.0
Other diseases of upper respiratory tract (J3O-J39)	30	11.7
Chronic lower respiratory diseases (J4O-J47)	13	5.1
Other diseases of the respiratory system (J6O-J99)	3	1.2

ICD-10 = International Classification of Diseases 10th revision.

The prevalence of two or more sick leaves in a year was not associated with the age group of the employee by the chi-square test (p = 0.570), so the PR was not calculated for this variable. Table 5 shows the data on associations and PR.

**Table 5:** Association between the number of sick leaves of employees of the SESAU of Chapecó, SC, Brazil, and the exploratory variables using the chi-squared test and crude PR for statistically significant associations between 2015 and 2018

Variables	One sick leave	Two or more sick leaves	p-value	PR^*^ (95%CI)
	%(95%CI)	% (95%CI		
Sex				
Male	69.4 (626-76.2)	30.6 (23.8-37.3)	0.017	1
Female	60.3 (57.8-62.7)	39.7(37.3-42.2)		1.30 (0.99-1.71)
Age group (years)				
Upto 29	65.8(59.5-72.1)	34.2 (27.9-40.5)	0.570	Not statistically
30-39	61.7 (57.6-65.7)	38.3(34.3-42.3)		significant
40-49	59.9 (55.7-64.1)	40.1 (35.9-44.3)		
50-59	59.2 (53.8-64.7)	40.8(35.3-46.2)		
60 or older	62.3(49.8-74.8)	37.7 (25.2-50.2)		
Length of service (years)				
Up to 5	62.5 (59.3-65.7)	37.5 (34.3-40.7)	0.001	1
6-10	64.9 (60.2-69.7)	35.1 (30.3-39.8)		0.93 (0.77-1.14)
11-20	58.2(52.6-63.6)	41.8(36.3-47.3)		1.12 (091-1.37)
21 or more	44.0 (34.1-53.9)	56.0 (46.1-65.9)		1.49 (1.12-1.98)^*^
Annual sick leave (days)				
3-9	85.8(83.3-88.2)	14.2 (11.8-16.6)	< 0.001	1
10-30	48.1 (43.8-52.4)	51.9 (47.6-56.2)		3.65 (2.93-4.53)^*^
31-365	25.5(21.0-30.0)	74.5 (70.0-79.0)		5.24 (4.21-6.51)^*^

95%CI = 95% confidence interval; SESAU = Secretaria Municipal de Saúde. Chi-squared test significant p-value ≤ 0.05.

* Statistically significant prevalence ratio (PR) (p ≤ 0.05)

Although the longest length of service (21 years or more) had the lowest frequency of sick leave (5.9%), the prevalence for this group was 49% higher than for those with up to 5 years of service, when considering that they had been on sick leave twice or more in the year. Longer periods of sick leave were also strongly associated with two or more sick leaves per year.

## DISCUSSION

The study analyzed official data identifying 1,695 professionals on sick leave for more than 3 days in the municipality of Chapecó, SC, from 2015 to 2018. The predominance of women, with 89.40% of cases, is in line with a study conducted in the municipality of Vitória, ES, which found that women represented 81.02% of sick leave.^9^ Another study found that 72.08% of sick leave was granted to women, after investigating the granting of sick leave to health workers in the Distrito Federal.^10^ The authors also reported similar results in a study in Goiânia, GO, which found that women accounted for 76.10% of sick leave.^11^

This higher rate among women may be related to the fact that women are predominantly healthcare workers; however, as women often work a double shift, involving household chores, responsibility for childcare, and their actual job, this can result in mental and physical strain, which contributes to a higher prevalence of absenteeism among women.^5,6,12^

As for length of service, it is noteworthy that 52.66% of sick leave occurred among people with less than 5 years in their careers. This prevalence was also evidenced in Leão et al.^11^ and Bastos et al.,^9^ who observed a higher occurrence of sick leave among workers in the first years of their careers.

The reasons for the increase in sick leave among workers with a shorter length of service may be associated with a deficit in the expert assessment at the time of admission, a lack of experience and knowledge on the job, failures in training programs, poor support from the supervisor, and difficulties in adapting quickly to new duties. These factors can expose workers to a high level of work-related stress, leading to an increase in sick leave as an alternative to coping with workrelated stress.^13^

A study in Ireland found that younger and less experienced health professionals experience more stressful situations, as they are less confident in making decisions, less able to control their emotions, and less organized in the face of work demands when compared to older and more experienced workers.^14^

A higher PR of having two or more sick leaves per year among civil servants with longer lengths of service (21 years or more) when compared to those with less than 5 years, is in line with a study including civil servants in municipalities of Goiânia and Vitória.^11,15^ The natural ageing of organic structures associated with a longer period of exposure to occupational risk factors may explain the 49% higher prevalence of two or more sick leaves per year among civil servants with longer lengths of service.

As for the clinical causes of sick leave, diseases in Chapter XIII, of the musculoskeletal system and connective tissue, accounted for 21.8% of cases. This finding is in line with Rocha et al.,^16^ Lucca & Rodrigues,^12^ and França et al.,^17^ which found a prevalence ranging from 17.8 to 23.6%.

Musculoskeletal disorders account for 53% of all occupational illnesses reported in the European Union and 50% of cases that lead to sick leave for more than 3 days. In Brazil, according to Social Security records, musculoskeletal disorders were responsible for the highest prevalence of sickness benefits throughout the last decade.^18^

Almeida et al.^19^ found that diseases of the musculoskeletal system and connective tissue were the main wear and tear disorder reported by CHWs, accounting for 10.66% of all ICD-10 disease groups. This information may be one of the reasons why this category had the highest prevalence of sick leave among all the other workers in this study.

According to the World Health Organization,^20^ workrelated musculoskeletal disorders (WMSDs) are due to excessive use of some part of the musculoskeletal system as a result of work-related physical activities which cause different degrees of functional incapacity, ranging from mild and transient disorders to irreversible and disabling injuries.

Musculoskeletal damage is defined as a set of conditions resulting from inflammation or degeneration of tendons, nerves, and ligaments, which can affect any region of the locomotor system. Among the main structures affected are painful disorders of the spine, generically known as lumbago and dorsalgia.^21^ This study also highlighted these disorders as among the subgroups of diseases of the musculoskeletal system.

This study found that dorsopathies had the highest prevalence, accounting for 57.6% of the causes of sick leave in the subgroup of musculoskeletal diseases. This disease also predominated in a study on absenteeism conducted at the Oswaldo Cruz Foundation, which found that dorsopathies accounted for 48.8% of musculoskeletal absences.^22^ According to Leão et al.,^11^ “this dysfunction, predominantly of mechanical origin, is generally preventable at a primary level, through the use of simple resources such as health education.”

Dorsalgias are part of WMSDs. They represent a health problem for workers, with economic and social impacts, especially when associated with functional incapacities, affecting their productive capacity and preventing them from working. Social Security data from 2013, based on notifications of work-related diseases, showed that dorsalgia was the third most frequent diagnosis in absolute numbers and the main diagnosis of back pain that led to disability pensions.^23^

As for the diagnoses that led to sick leave, mood/ depressive disorders were the second most significant cause in our study. This subgroup of illnesses is responsible for the main mental disorders in the population. It is estimated that approximately 350 million people, or 5% of the world’s population, suffer from depression, and in the coming years this will be the biggest cause of disability in the world. In Brazil, depression affects 10% of the population.^24^

Depression, which is considered to be the disease of the century, affects all professionals; however, it most frequently affects health professionals. Among these professionals, those in the medical and nursing professions and CHWs are the categories most exposed to mental suffering.^25^

This study had a number of limitations. There was a loss of information for the third quarter of 2018, as the data was not fully consolidated at the time of collection, compromising a more reliable analysis of sick leave. It was also not possible to access information on the total number of active civil servants at SESAU during the years studied, which made it impossible to estimate the representation of sick leaves among all civil servants and some absenteeism indicators that would allow comparison with other scenarios studied.

## CONCLUSIONS

This study provided an overview of sickness absenteeism among public health workers in the municipality of Chapecó between 2015 and 2018. The demographic profile of absenteeism among civil servants was CHWs, women, mean age of 30 to 39 years, with the majority having one absence per year and a total of 3 to 9 days.

We recommend that further studies be conducted with this population, focusing on the professionals’ perception of their health, working conditions, organizational model, and motivation to work.

This study allowed us to broaden our knowledge of the pattern of sickness absenteeism among workers in Chapecó health network. These results will allow for the development of HR management indicators, or may even be used as such, as well as fostering strategies to promote healthy environments, prevent illnesses and diseases, and rehabilitation.
